# Molecular docking unveils the potential of andrographolide derivatives against COVID-19: an in silico approach

**DOI:** 10.1186/s43141-022-00339-y

**Published:** 2022-04-14

**Authors:** Ravichandran Veerasamy, Rohini Karunakaran

**Affiliations:** 1 Pharmaceutical Chemistry, Faculty of Pharmacy, AIMST University, 08100 Semeling, Malaysia; 2 Saveetha Dental College and Hospitals, Saveetha Institute of Medical and Technical Sciences, Chennai, Tamil Nadu India; 3 Centre of Excellence for Biomaterials Science, AIMST University, 08100 Semeling, Bedong Malaysia; 4 Faculty of Medicine, AIMST University, 08100 Semeling, Kedah Malaysia

**Keywords:** SARS-CoV-2, COVID-19, Andrographolide, Molecular docking, Pharmacokinetics, Drug−likeness profiles

## Abstract

**Background:**

The recent severe acute respiratory syndrome coronavirus-2 (SARS-CoV-2) infection cause high mortality and there is an emergency need to develop a specific drug to treat the novel coronavirus disease, COVID-19. However, some natural and synthetic products with action against SARS-CoV-2 have been reported in recent research, there is no specific drug available for treating COVID-19. In the present study, molecular interaction analysis was performed for 16 semisynthetic andrographolides (AGP) against 5 SARS-CoV-2 enzymes main protease (Mpro, PDB: 6LU7), papain-like protease (PLpro, PDB: 6WUU), spike glycoprotein (S, PDB: 6VXX), NSP15 endoribonuclease (NSP15, PDB: 6VWW), and RNA-dependent RNA polymerase (RdRp, PDB: 6M71). Moreover, the compounds pharmacokinetic and toxic profiles were also analyzed using computational tools.

**Results:**

The protein−ligand docking score (kcal/mol) revealed that all the tested AGP derivatives showed a better binding affinity towards all the tested enzymes than hydroxychloroquine (HCQ). Meanwhile, all the tested AGP derivatives showed a better binding score with RdRp and S than remdesivir (REM). Interestingly, compounds 12, 14, and 15 showed a better binding affinity towards the all the tested enzyme than AGP, REM, and HCQ. AGP-16 had shown − 8.7 kcal/mol binding/docking score for Mpro, AGP-15 showed − 8.6 kcal/mol for NSP15, and AGP-10, 13, and 15 exhibited − 8.7, − 8.9, and − 8.7 kcal/mol, respectively, for S.

**Conclusion:**

Overall results of the present study concluded that AGP derivatives 14 and 15 could be the best ‘lead’ candidate for the treatment against SARS-CoV-2 infection. However, molecular dynamic studies and pharmacological screenings are essential to developing AGP derivatives 14 and 15 as a drug against COVID-19.

**Supplementary Information:**

The online version contains supplementary material available at 10.1186/s43141-022-00339-y.

## Background

In the twenty-first century, coronavirus disease−2019 (COVID-19), one of the most destructive pandemics, was identified after an abnormal pneumonia outbreak in Wuhan, China, caused by severe acute respiratory syndrome coronavirus-2 (SARS-CoV-2) [1]. It generated a global health emergency by its rapid transformation and higher mortality and disrupted the normal human life [2]. The WHO reports > 233 million infected cases and > 4.7 million deaths from SARS-CoV-2 until 1st October 2021 [1]. Main protease (Mpro), papain-like protease (PLpro), spike glycoprotein (S), RNA-dependent RNA polymerase (RdRp), envelop protein (E), membrane protein (M), and nucleocapsid (NSP) are some essential proteins of the virus, particularly SARS-CoV-2 that are responsible for virus interaction with the host, and replication of virus genomes in the host cell [3–5]. However, human angiotensin-converting enzyme 2 (ACE2) play an essential role in viral entry and fusion [5]. These are the main targets to treat viral infections.

Andrographolide (AGP) is a significant bioactive phytoconstituent present in various *Andrographis paniculata*, family Fabaceae. Chemically andrographolide is 3α, 14, 15, 18-tetrahydroxy-5β, 9βH, 10α-labda-8, 12-dien-16-oic acid γ-lactone, with molecular formula C_20_H_30_O_5_. The reported pharmacological effects of AGP are neuroprotective, immunostimulatory, antioxidant, anti-cancer, anti-inflammatory, anti-microbial, anti-hepatotoxic, and anti-viral to treat dengue chikungunya, swine flu, influenza. AGP is also found to be used in the treatment of upper respiratory tract infections [6]. Thailand announced its pilot program for administering and examining the efficacy of *A. paniculata* extract in COVID-19 patients [7]. *Xiyanping*, a Traditional Chinese Medicine containing andrographolide as one of its ingredients, has been recommended for COVID-19 in China under the China National Health Commission treatment guidelines [8]. Shi et al. (2020) reported that the 3CLpro inhibitory activity of andrographolide was lower than disulfiram [9]. Another Vero cell-based study reported potential SARS-CoV-2 inhibitory activity for both andrographolide and *A. paniculata* extract [10].

The versatility of AGP as a SARS-CoV-2 anti-viral is demonstrated by its potential to bind to several important targets (spike protein-ACE-2 receptor complex, spike protein, ACE-2 receptor, RdRp, 3CLpro, PLpro, and N-protein RNA-binding domain) at various stages of viral attachment, replication, and host-pathogen interactions [9, 11–19]. This property may be a vital lead to any potential therapeutic agent being developed. Molecular dynamics study of AGP with SARS-CoV-2 suggests that AGP can bind to both SARS-CoV-2 spike protein and human ACE-2 receptors [11, 15]. There was no andrographolide or any compound of *A. paniculata* in the top 6 potential hits when an in silico and in vitro high throughput screening of 10,000 compounds containing natural compounds was conducted to identify potent 3CLpro inhibitors. Some researchers reported that andrographolide and its natural analogues had less binding affinity than remdesivir or other phytochemicals such as curcumin. However, andrographolide and related compounds were predicted to have binding affinity to several key components of the SARS-CoV-2 life cycle and pathogenicity [9, 11, 13–19]. In addition, andrographolide and the major bioactive compounds of *A. paniculata* may have additional pleiotropic effects on post-viral infection due to their reported anti-inflammatory and immunomodulatory properties [20–25].

Exploring analogues of andrographolide as antivirals may be helpful as these compounds have been suggested to have better binding affinities towards viral targets than the parent andrographolide structure. To date, no specific medications have been developed. Therefore, considering the risk factors associated with this disease, there is an urgent need for a treatment method to treat this disease to limit the transmission [26]. Artificial intelligence plays a crucial role in drug design and discovery to reduce time and cost, mainly used in the repurposing of FDA approved drugs and to find the application of natural, synthetic, and semisynthetic derivatives in the treatment of various diseases, disorders, and infections. Hence, we have done docking studies on some semisynthetic and designed AGP derivatives, with hypothesized that the semisynthetic and designed AGP derivatives would have better interaction affinity towards the main targets of COVID-19 disease than AGP, and remdesivir and hydroxychloroquine, the drugs that are showing better affinity against COVID-19 disease targets. All the reported semisynthetic and a few of our designed AGP derivatives showed better affinity than AGP and used drugs. This study provides some preliminary scientific evidence for the use of AGP derivatives to combat SARS-CoV-2. However, further in vitro and in vivo experimental studies need to be done to confirm the anti-COVID action potential of AGP derivatives.

## Methods

The modelling software Chem Office-16 (http://www.cambridgesoft.com/Ensemble_ for_Chemistry/details/Default.aspx?fid=16), Discovery Studio Visualizer 3.0 (https:// discover.3ds.com/discovery-studio-visualizer-download), Swiss Protein Data Base Viewer (https://spdbv.vital-it.ch/), Open Babel (http://openbabel.org/wiki/Main_Page), PyRx (https://pyrx.sourceforge.io/), AutoDock Vina (http://vina.scripps.edu/) and online tool Swiss ADME (http://www.swissadme.ch/), ProTox-II (https://tox-new.charite.de/protox_II/) was used in the study. The 3D structures of SARS-CoV-2 proteins such as main protease (Mpro, PDB: 6LU7), papain-like protease (PLpro, PDB: 6WUU), spike glycoprotein (S, PDB: 6VXX), NSP15 endoribonuclease (NSP15, PDB: 6VWW), and RNA-dependent RNA polymerase (RdRp, PDB: 6M71) were downloaded from Protein Data Bank.

### Preparation of ligands

AGP structures were drawn using the Chem Draw tool, converted to 3D structure using the Chem3D tool in Chem Office-16, and saved as either .sdf or .mol file for further use.

### In silico physicochemical and ADME/T studies

Physicochemical, pharmacokinetic and toxicity of AGP derivatives were calculated using online tools SwissADME and ProTox-II.

### Active site predictions

The active binding site of the SARS-CoV-2 proteins were predicted, 5 Å sphere was generated from the center of the binding site, and the coordinate or the spheres x, y, and z centres were calculated using Discovery studio visualizer for the crystallographic structure of protein complex with ligand. For the apoprotein, a blind docking procedure was adopted, the total protein structure was considered to create a grid box.

### Preparation of proteins

The crystallographic structures of SARS-CoV-2 proteins were checked for broken chain and errors using Swiss Protein Data Base Viewer and corrected. Then, the water and other heteroatoms were removed, polar hydrogens were added to the protein structure, charges assigned, and saved as PDB format for docking study [27].

### Docking studies

AGP derivatives and protein structures were uploaded in the Virtual Screening software interface PyRx 0.8 utilizes Autodock Vina and Autodock 4.2. These structures were then energy-minimized using the conjugate gradient optimization algorithm (200 iterations) with MMFF94 forcefield. Local search method Broyden-Fletcher-Goldfarb-Shanno (BFGS) used to energy minimization of protein. Then, the protein and ligand molecules were converted to the ‘.pdbqt’ format using auto dock tools. The active binding site grid box was generated automatically by PyRx. The size and coordinate of grid box were adjusted by entering the calculated values from discovery studio visualizer in the appropriate box [27]. Blind docking was carried out for RNA-dependent RNA polymerase and spike glycoprotein (closed), since the electron microscopic structure of these proteins are available as apoproteins. All other software parameters were kept as default and ligand were considered as flexible and receptor as rigid. PyRx uses Lamarckian genetic algorithm as scoring function. The final visualization of the docked structure was performed using Discovery Studio Visualizer 3.0. The best conformer was selected based on the docking score and better non-covalent bond interaction. The docking pose and interactions pictures were collected and saved [28].

## Results

**Fig. 1:**
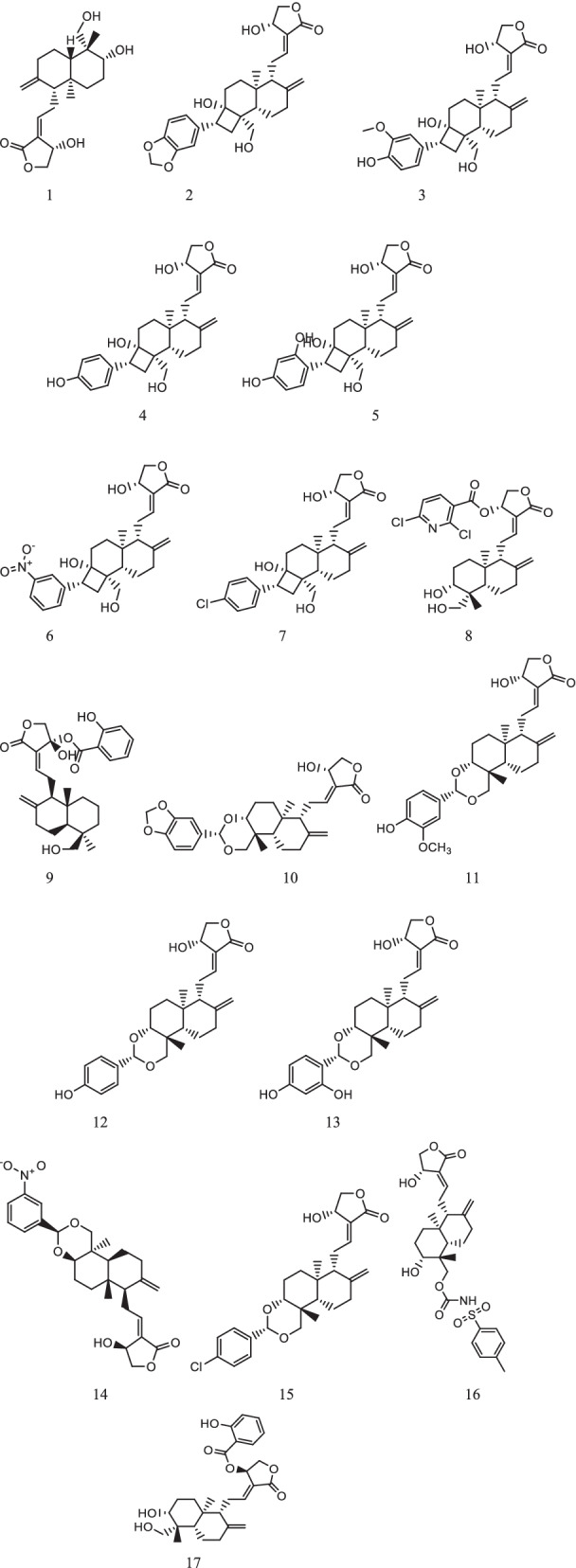
Structures of AGP derivatives

**Fig. 2:**
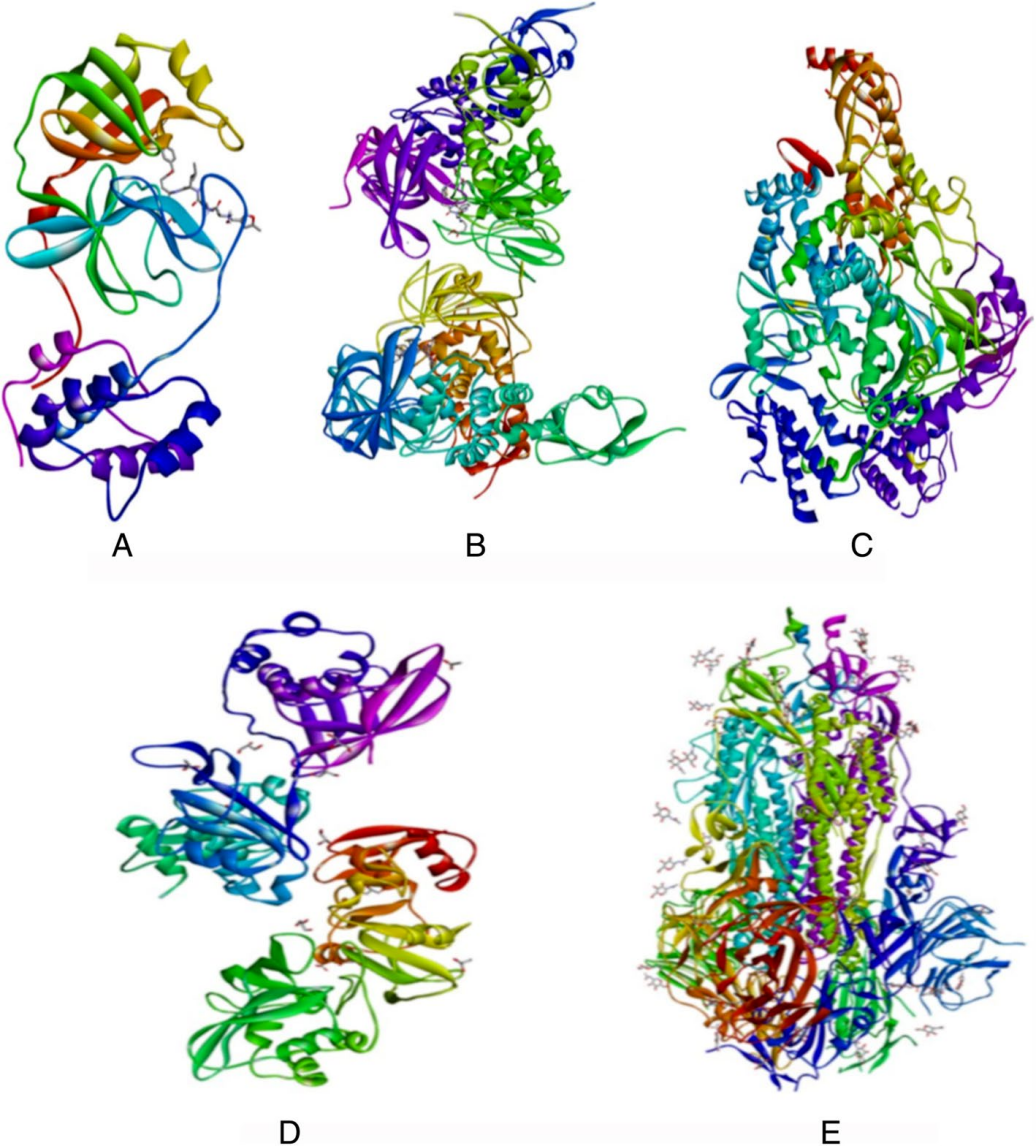
Structures of SARS-CoV-2 proteins. **A** Mpro. (PDB: 6LU7). **B** PLpro (PDB: 6WUU), **C** Spike glycoprotein (PDB: 6VXX). **D** NSP15 endoribonuclease (PDB: 6VWW). **E** RNA-dependent RNA polymerase (PDB: 6M71)

In the present research work, molecular interactions of AGP derivatives with SARS-CoV-2 viral proteins were studied. In this study, we used a total of 17 semisynthetic AGP derivatives (Fig. 1) with andrographolide, remdesivir, and hydroxychloroquine. In the selected AGP derivatives, nine were taken from the earlier reported study [29] and eight were knowledge based designed compounds. PDB structures of RdRp, PLpro, Mpro, NSP15, and spike protein were given in Fig. 2.

### Physicochemical and toxicity properties

The SwissADME web tool was used to compute the physicochemical properties, which was used to identify the drug-likeness of AGP derivatives. The compounds that satisfied Lipinski rule of five for drug-likeness was considered for further study. The physicochemical and pharmacokinetic properties of the AGP derivatives were depicted in Table 1. The AGP derivatives toxicity profile was predicted using online tool ProTox-II, and the toxicity details were tabulated in Table 2.

**Table 1:** Drug likeness and pharmacokinetic properties of AGP derivatives

Compounds	MW	logP	HBA	HBD	Rotatable bond	TPSA	BBB permeant	GI Absorption	BA scores
1 AGP	350.45	1.96	5	3	3	86.99	No	High	0.55
2	482.57	3.23	7	3	4	105.45	No	High	0.55
3	484.58	3.21	7	4	5	116.45	No	High	0.55
4	454.56	3.21	6	4	4	107.22	No	High	0.55
5	470.56	2.91	7	5	4	127.45	No	High	0.55
6	483.55	3.93	7	3	5	132.81	No	Low	0.55
7	473.00	4.15	5	3	4	86.99	No	High	0.55
8	524.43	4.52	7	2	6	105.95	No	High	0.55
9	470.55	3.88	7	3	6	113.29	No	High	0.55
10	482.50	4.45	7	1	3	83.47	No	High	0.55
11	484.59	4.44	7	2	4	94.46	No	High	0.55
12	454.56	4.43	6	2	3	85.23	No	High	0.55
13	470.56	4.13	7	3	3	105.46	No	High	0.55
14	483.56	5.16	7	1	4	110.83	No	High	0.55
15	473.01	5.38	5	1	3	65.00	Yes	High	0.55
16	547.67	4.87	8	3	8	147.61	No	Low	0.55
17	470.56	3.53	7	3	6	113.29	No	High	0.55
REM	602.58	3.28	12	4	14	213.36	No	Low	0.17
HCQ	335.87	3.35	3	2	9	48.39	yes	High	0.55

*MW* molecular weight, *HBA* hydrogen bond acceptor, *HBD* hydrogen bond donor, *TPSA* topological polar surface area, *AGP* andrographolide, *REM* remdesivir, *HCQ* hydroxychloroquine

**Table 2:** Toxicity profile of AGP derivatives

Compounds	LD_**50**_ score (mg/kg)	Hepato-toxicity	Carcino-genecity	Immuno toxicity	Muta-genicity	Cyto-toxicity	Toxicity class
1 AGP	1890	Safe	Safe	Toxic	Safe	Mod.Secure	4
2	1050	Safe	Safe	Toxic	Safe	Mod.Secure	4
3	1050	Safe	Safe	Toxic	Safe	Safe	4
4	2000	Safe	Safe	Toxic	Safe	Mod.Secure	4
5	2000	Safe	Safe	Toxic	Safe	Mod.Secure	4
6	2000	Mod.Secure	Mod.Secure	Toxic	Mod.Safe	Mod.Secure	4
7	2000	Safe	Safe	Toxic	Safe	Mod.Secure	4
8	10000	Safe	Safe	Toxic	Mod.Secure	Mod.Secure	6
9	620	Safe	Safe	Toxic	Safe	Safe	4
10	2000	Safe	Mod.Secure	Toxic	Safe	Safe	4
11	2500	Safe	Mod.Secure	Toxic	Safe	Safe	5
12	590	Safe	Mod.Secure	Toxic	Safe	Mod.Secure	4
13	590	Safe	Mod.Secure	Toxic	Safe	Mod.Secure	4
14	590	Mod.Secure	Mod.Safe	Toxic	Mod.Safe	Mod.Secure	4
15	2000	Safe	Mod.Secure	Toxic	Safe	Mod.Secure	4
16	1000	Mod.Secure	Mod.Secure	Safe	Mod.Secure	Mod.Secure	4
17	620	Safe	Safe	Toxic	Safe	Safe	4
REM	1000	Mod.Secure	Mod.Secure	Safe	Mod.Secure	Mod.Secure	4
HCQ	1240	Safe	Mod.Secure	Toxic	Toxic	Safe	4

*AGP* andrographolide, *REM* remdesivir, *HCQ* hydroxychloroquine

### Active site

The active binding site of the SARS-CoV-2 proteins was predicted using Discovery studio visualizer for the crystallographic structure of protein complex with ligand, and the coordinate/center of the *x*, *y*, and *z* axes of the spheres of each protein active binding site was given in Table 3. A blind docking procedure was adopted for the apoprotein.

**Table 3:** Coordinate or the *x*, *y*, and *z* centers of grid boxes of breast cancer proteins with dimensions

Protein molecule	***x*** centers (dimension)	***y*** centers (dimension)	***z*** center (dimension)
Mpro	− 10.86 (13 Å)	12.02 (13 Å)	69.07 (13 Å)
PLpro	23.81 (12 Å)	61.31 (12 Å)	− 12.78 (12 Å)
RdRp	116.16 (15 Å)	116.37 (15 Å)	136.62 (15 Å)
NSP15	− 91.15 (25 Å)	21.86 (25 Å)	− 30.63 (25 Å)
S	199.41 (61 Å)	229.04 (61 Å)	241.30 (61 Å)

### Docking study

The present docking study was validated by re-docking extracted ligand from the crystallographic protein-ligand complex structure with the same protein. All binding conformations of the re-docked ligand within the binding pocket of protein produced by the PyRx 0.8 tool were like the binding mode of the co-crystallized ligand, and the root means square deviation (RMSD) for these conformations were below 2 Å. The docking method for NSP15 (PDB: 6VWW) and RNA-dependent RNA polymerase (PDB: 6M71) was validated by repeated docking with remdesivir. The output was the same for multiple times of docking means the interaction took place in the same site with the identical residues, and the root means square deviation (RMSD) for these conformations were below 2 Å, hence the docking studies for AGP derivatives were carried out using PyRx 0.8 tool. The binding affinity/energy of the AGP derivatives towards SARS-CoV-2 proteins were given in Table 4, and the details of interacting amino acids of proteins with AGP derivatives with the highest binding energy were given in Table 5.

**Table 4:** Binding affinity of AGP derivatives with protein targets of SARS-CoV-2

S. No	Compounds	Binding affinities (kcal/mol)				
		Mpro	PLpro	RdRp	NSP15	S
1	1 (AGP)	− 6.6	− 6.8	− 6.4	− 6.7	− 7.1
2	2	− 7.8	− 8.0	− 7.8	− 6.6	− 8.0
3	3	− 7.4	− 7.6	− 7.6	− 8.3	− 7.3
4	4	− 7.3	− 7.4	− 7.5	− 7.9	− 8.2
5	5	− 7.4	− 7.6	− 7.6	− 7.8	− 7.0
6	6	− 7.7	− 7.9	− 8.1	− 7.8	− 7.6
7	7	− 7.3	− 7.6	− 7.2	− 8.1	− 8.4
8	8	− 7.8	− 7.9	− 7.9	− 8.1	− 7.0
9	9	− 7.6	− 7.7	− 7.6	− 8.1	− 7.6
10	10	− 8.2	− 7.8	− 8.3	− 8.4	− 8.7
11	11	− 8.3	− 7.3	− 8.0	− 7.7	− 8.1
12	12	− 7.9	− 7.9	− 7.9	− 8.2	− 8.1
13	13	− 8.3	− 7.4	− 8.4	− 8.1	− 8.9
14	14	− 8.2	− 8.1	− 8.5	− 8.1	− 8.4
15	15	− 7.9	− 7.8	− 7.9	− 8.6	− 8.7
16	16	− 8.7	− 7.2	− 7.7	− 7.6	− 7.7
17	17	− 8.1	− 7.6	− 6.9	− 7.6	− 7.7
18	REM	− 7.8	− 7.4	− 6.1	− 6.7	− 7.0
19	HCQ	− 6.2	− 5.7	− 5.3	− 5.9	− 5.9

*AGP* andrographolide, *REM* remdesivir, *HCQ* hydroxychloroquine

**Table 5:** Amino acids of SARS-CoV-2 protein involved in interactions with AGP derivatives of high binding energy

Protein	Compound	Amino acids involved in the interaction		
		Hydrogen bond	Hydrophobic	Others
Mpro	REM	Gly143, Ser144 (2), Cys145, Glu166, Asn142	Met165, His41	Gln189 (C), Glu166 (C)
	HCQ	Thr190	His41, Met49, Cys145	Glu166 (C)
	AGP	Leu141, Met165	–	–
	16	Thr24	Met49 (2), His41, Met165	Met165 (C)
	11	Gly143, Ser144, Glu166, Gln189	Cys145	–
PLpro	REM	Gly163, Asp164	Pro248	Asp164 (E), Pro248 (C), Tyr268 (C)
	HCQ	Ala246, Tyr273, Asp302	Leu162, Tyr264	Asp164 (E), Ser245 (C)
	AGP	Tyr264, Gly271		
	14	Leu162, Tyr273	Tyr264, Pro248	Glu161 (C)
	2	Lys157, Arg166 (2), Thr301, Tyr268	–	Ala246 (C), Glu167 (C)
RdRp	REM	Tyr619, Asp760, Trp617	Lys798 (2)	Asp760 (E), Pro620 (C)
	HCQ	Ala840, Phe843, Arg858	Ala547, Ile548, Arg858	Asp845 (C)
	AGP	Lys621, Tyr619, Asp760, Glu811	–	–
	14	Lys621, Ser795 (2), Lys798, Ser814	Pro620	Pro620 (C), Ser814 (C)
	13	Trp617, Asp761	Lys621, Arg624	Arg553 (E), Asp623 (E)
	6	Ser795, Cys813, Ser814	–	Asp761(E), Glu811 (E)
NSP15	REM	Thr341, Gln245	Trp333 (2), Val292, Lys345, Leu346, Tyr343	–
	HCQ	Val276, Lys277, Asn278	Ile270, Val276, Ile328 (2)	–
	AGP	Gly248, Lys290, His235	Tyr343	Lys345 (C)
	15	Ser294 (2)	Lys290, Val315, Trp333	His250 (C)
	3	Lys290 (2), Leu346	Thr341, His235	Lys345 (C), Glu340 (C)
S	REM	Gly744, Asn856, Leu977, Arg1000 (2), Phe855, Asp745	Val963, Lys964 (2)	Met740 (C), Val963 (C), Lys964 (C)
	HCQ	Asp364, Cys336	Trp436	Ala363 (C), Asp364 (C)
	AGP	Lys206	Pro225, Tyr38	Phe43 (C)
	13	Arg190, His207	Leu226, Val227, Val126	–
	15	Arg190 (2), Asn121	Val126, Leu226, Val227, Tyr170, Phe168	–
	7	Arg1000 (2), Asn856, Leu966	Val963, Leu966	–

*E* electrostatic, *S* pi-sulfur bond, *C* carbon-hydrogen bond

Numerical value in the parenthesis next to amino acid residue indicates the number of interactions of that residue

**Fig. 3:**
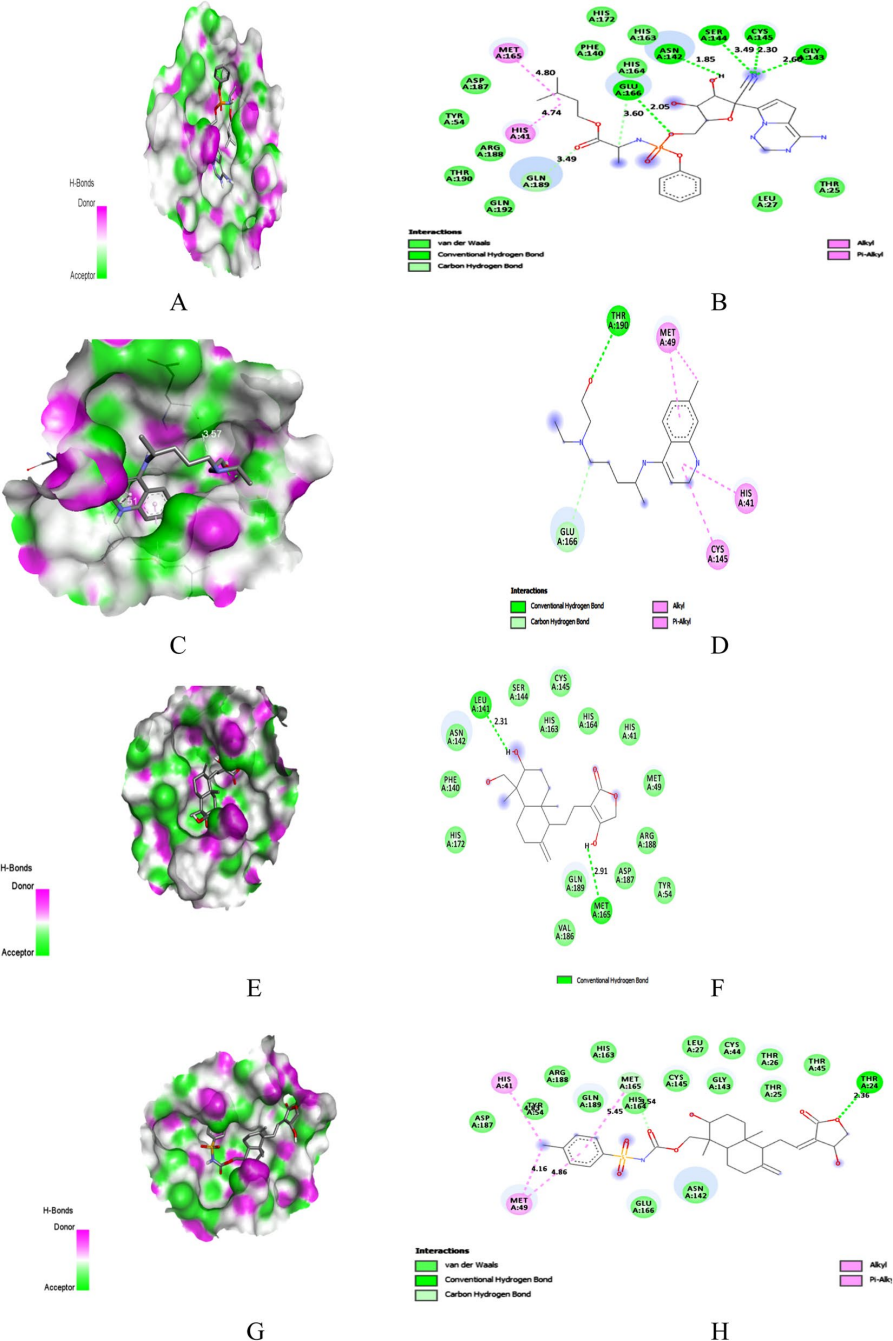
Docking with Mpro. **A** Docking pose of REM on hydrogen bonding surface. **B** 2D interaction of REM. **C** Docking pose of HCQ on hydrogen bonding surface. **D** 2D interaction of HCQ. **E** Docking pose of AGP on hydrogen bonding surface. **F** 2D interaction of AGP. **G** Docking pose of AGP-16 on hydrogen bonding surface. **H** 2D interaction of AGP-16

**Fig. 4:**
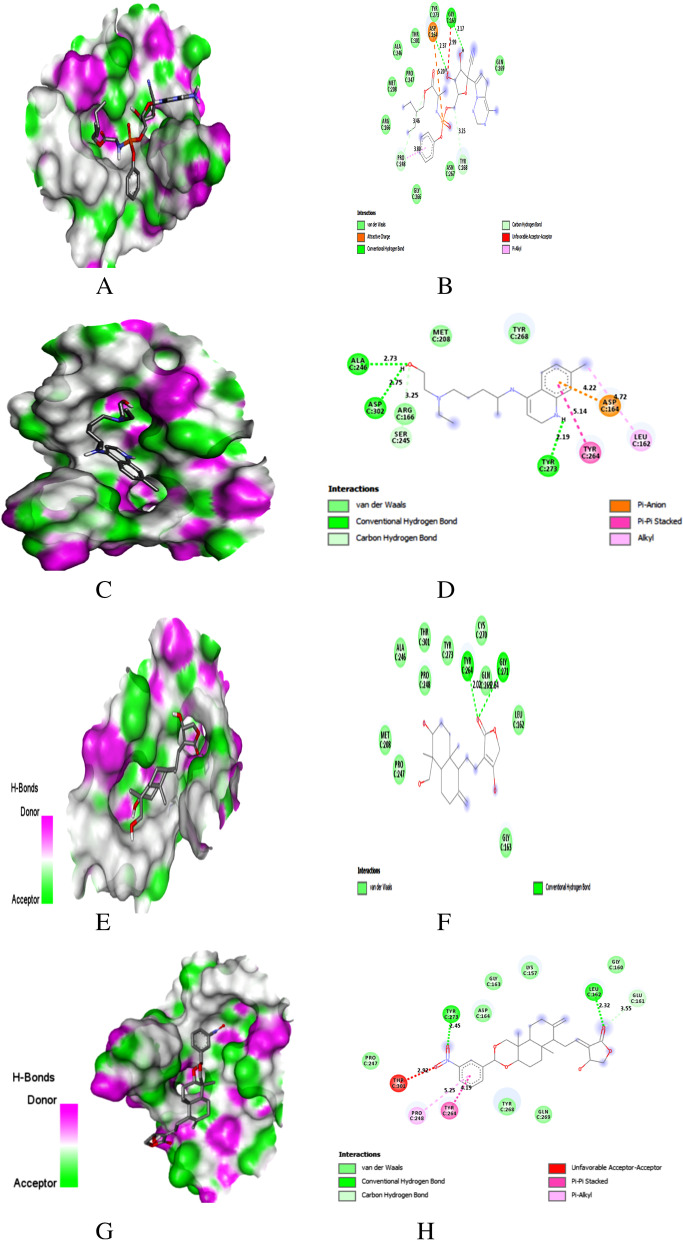
Docking with PLpro. **A** Docking pose of REM on hydrogen bonding surface. **B**) 2D interaction of REM. **C** Docking pose of HCQ on hydrogen bonding surface. **D** 2D interaction of HCQ. **E** Docking pose of AGP on hydrogen bonding surface **F** 2D interaction of AGP. **G** Docking pose of AGP-14 on hydrogen bonding surface. **H** 2D interaction of AGP-14

**Fig. 5:**
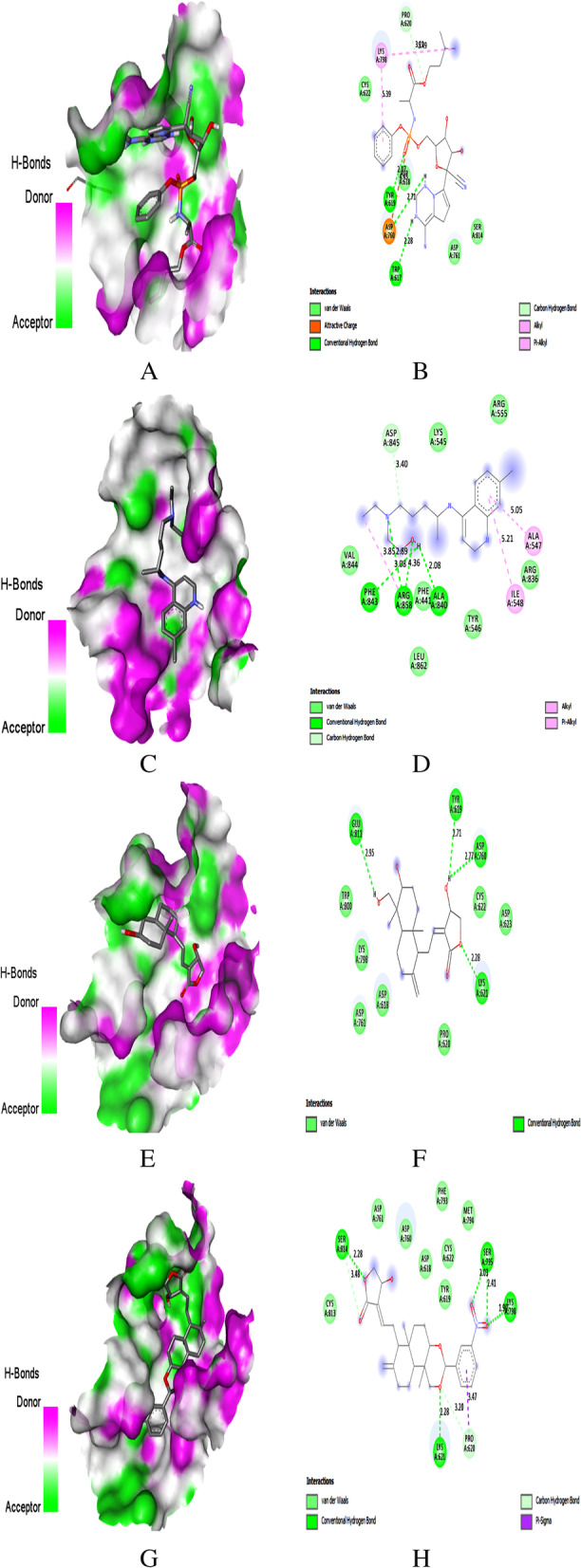
Docking with RdRp. **A** Docking pose of REM on hydrogen bonding surface. **B**) 2D interaction of REM. **C** Docking pose of HCQ on hydrogen bonding surface. **D** 2D interaction of HCQ. **E** Docking pose of AGP on hydrogen bonding surface. **F** 2D interaction of AGP. **G** Docking pose of AGP-14 on hydrogen bonding surface **H** 2D interaction of AGP-14

**Fig. 6:**
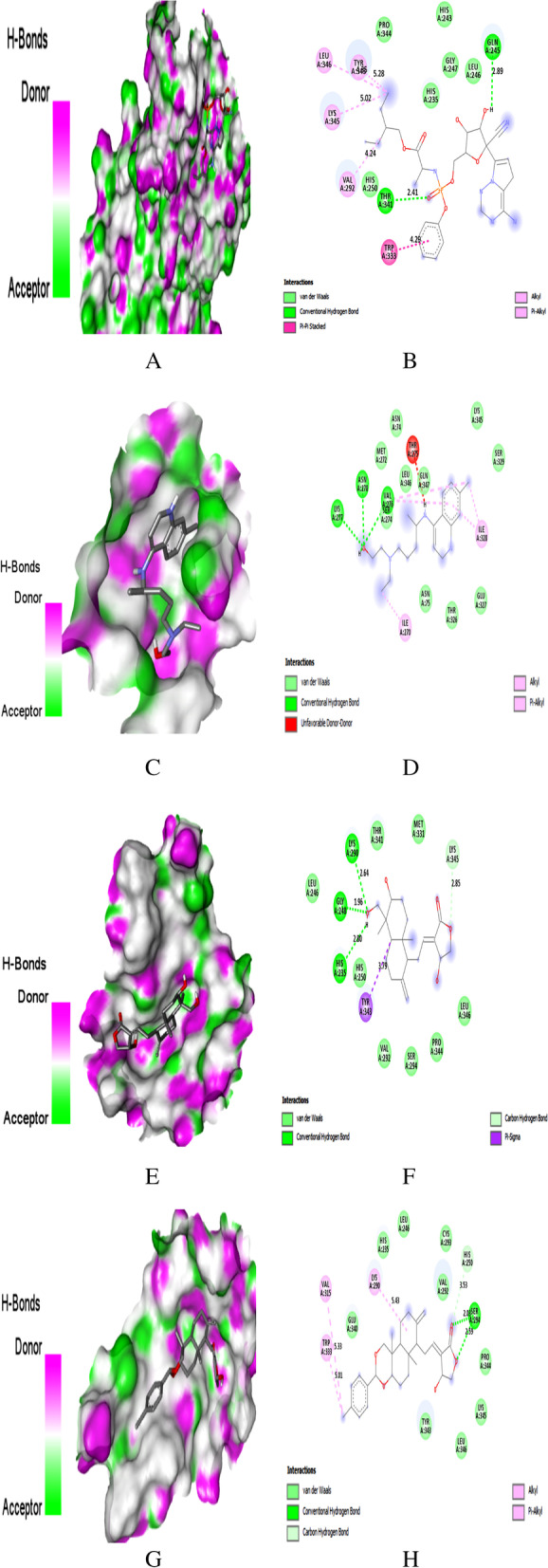
Docking with NSP15. **A** Docking pose of REM on hydrogen bonding surface. **B** 2D interaction of REM. **C** Docking pose of HCQ on hydrogen bonding surface. **D** 2D interaction of HCQ. **E** Docking pose of AGP on hydrogen bonding surface. **F** 2D interaction of AGP. **G** Docking pose of AGP-15 on hydrogen bonding surface. **H** 2D interaction of AGP-15

**Fig. 7:**
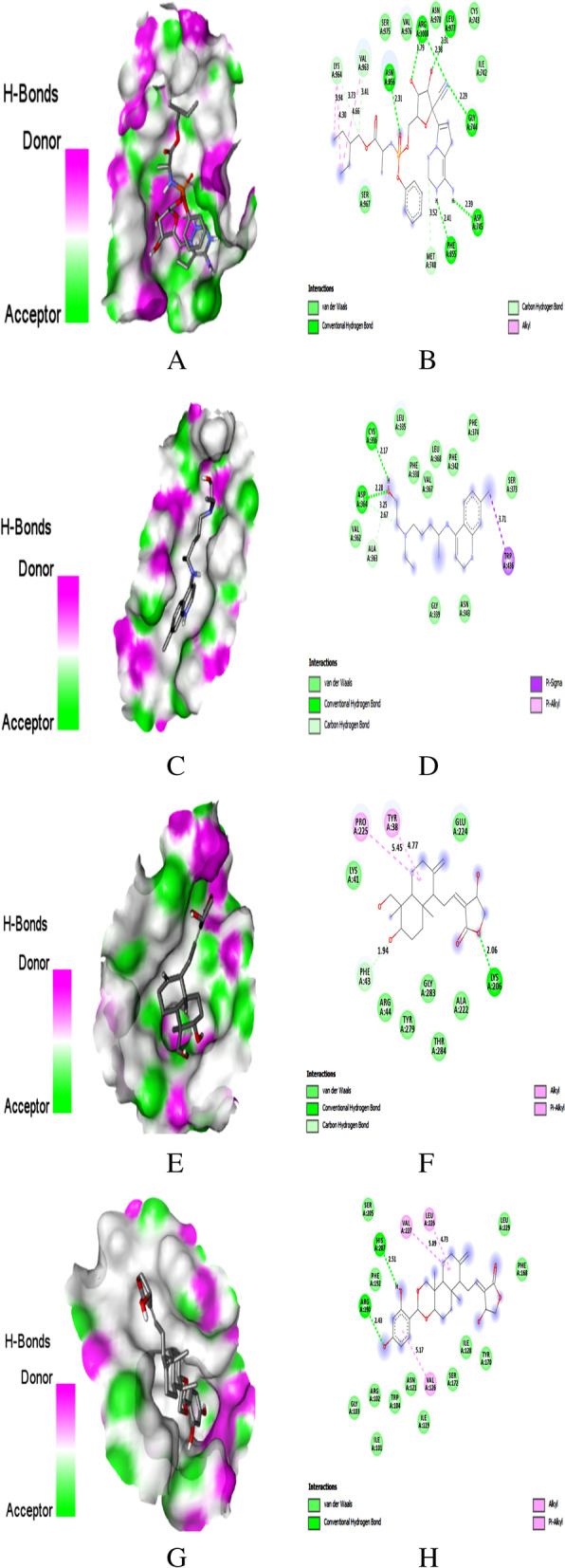
Docking with spike glycoprotein. **A** Docking pose of REM on hydrogen bonding surface. **B** 2D interaction of REM. **C** Docking pose of HCQ on hydrogen bonding surface. **D** 2D interaction of HCQ. **E** Docking pose of AGP on hydrogen bonding surface. **F** 2D interaction of AGP, **G** Docking pose of AGP-13 on hydrogen bonding surface. **H** 2D interaction of AGP-13

The interacting amino acids of SARS-CoV-2 proteins with all AGP derivatives and reference drugs were given in Table S1 as supplementary data. The PyRx docking output ‘.pdbqt’ files of AGP were analyzed in the discovery studio visualizer. The 3D interaction, docking pose with hydrogen bond donor surface, and 2D interaction image of docking of AGP derivatives on SARS-CoV-2 proteins were generated using a discovery studio visualizer. The docking pose and interaction images of REM, HCQ, and AGP derivatives have the highest binding energy with Mpro, PLpro, RdRp, NSP15, and S were shown in Figs. 3, 4, 5, 6, and 7, respectively.

### Binding to Mpro

In-depth molecular docking analysis of AGP derivatives with the active sites of Mpro was performed to understand the protein-ligand interactions. Remdesivir and hydroxychloroquine were used as reference drugs to compare the interaction. Top-ranked conformations were selected based on the binding affinity to the reported binding channel of Mpro by hydrogen bonds and other non-covalent interactions.

REM and HCQ showed molecular interactions on the active site residue of Mpro with a binding score of − 7.8 and − 6.2 kcal/mol, respectively. The hydrogen bond interaction surface pose and 2D interaction of REM, HCQ, AGP, and AGP-16 on Mpro was shown in Fig. 2A, H. In REM complex, six H-bonds with Gly143, Ser144 (2), Cys145, Glu166, and Asn142, two hydrophobic and two C–H interactions (Fig. 3A, B) were observed while one H-bond at Thr190, three hydrophobic and a C–H interaction were observed in HCQ complex (Fig. 3C, D).

Meanwhile, AGP formed two H-bond interactions with residue Leu141 (2.31 Å) and Met165 (2.91 Å) of Mpro (Fig. 3E, F). AGP derivative 16 bound to the active site of Mpro with a binding score of − 8.7 kcal/mol while AGP with − 6.6 kcal/mol. AGP-16/Mpro complex showed that Thr24 (2.35 Å) was involved in H-bond formation, whereas hydrophobic interactions formed residues Met49, His41, and Met165. AGP-16 also showed C–H bond formation with Met165 (Fig. 3G, H). Two H-bonds, one hydrophobic, and a pi-sulfur interaction were noticed in the AGP-13/Mpro complex with − 8.3 kcal/mol (Table 4). However, AGP-11 also had a binding score of − 8.3 kcal/mol with four H-bonds at Gly143 (2.12 Å), Ser144 (2.46 Å), Glu166 (2.07 Å), Gln189 (2.58 Å), and one hydrophobic interaction at Cys145 on Mpro (Table 5).

### Binding to PLPro

Remdesivir and hydroxychloroquine were used as reference drugs to compare the interaction. Top-ranked conformations were selected based on the binding affinity to the reported binding channel of PLpro by hydrogen bonds and other non-covalent interactions.

The hydrogen bond interaction surface poses and 2D interactions of REM, HCQ, AGP and AGP-14 on PLpro were shown in Fig. 3A–H. REM is an anti-viral drug that showed the molecular interaction towards the active pocket residue of PLpro with a binding score of − 7.4 kcal/mol. Two H-bonds with Gly163, Asp164, one hydrophobic, two C–H, and one electrostatic interaction (Fig. 4A, B) were noticed in the REM/PLpro complex. While three H-bonds at Ala246, Tyr273, and Asp302, two hydrophobic, one electrostatic and a C–H interaction were observed for HCQ/PLpro complex with a binding score of − 5.7 kcal/mol (Fig. 4C, D).

AGP showed two H-bond interactions with residue Tyr264 (2.02 Å) and Gly271 (2.84 Å) of PLpro with a binding score of − 6.8 (Fig. 4E, F, Table 4). Whereas AGP derivative 14 showed that Leu162 (2.32 Å) and Tyr273 (2.45 Å) were involved in H-bond formation, residues Tyr264 and Pro248 were involved in hydrophobic interaction. Glu161 formed a C–H bond interaction with a − 8.1 kcal/mol (Fig. 4G, H, Tables 4 and 5). Five H-bonds at Lys157, Arg166 (2), Thr301, Tyr268, and two C–H bond interactions were noticed in the AGP-2/PLpro complex with a binding score of − 8.0 kcal/mol. However, AGP-6, 8, and 12 were shown a binding score of − 7.9 kcal/mol (Table S1).

### Binding to RdRp

REM and HCQ showed binding affinity against RdRp with − 5.3 and − 6.1 kcal/mol binding energy. Three H-bonds at residue Tyr619, Asp760, and Trp617, two hydrophobic, one electrostatic, and a C–H bond interaction were observed for REM/RdRp complex (Fig. 5A, B). Meanwhile, HCQ showed three H-bonds at Ala840, Phe843, and Arg858, three hydrophobic, and a C–H bond interaction with RdRp (Fig. 5C, D). All the studied AGP derivatives showed better binding energy with RdRp than REM and HCQ. AGP, AGP-14, 13, and 6 were shown binding energy of − 6.4, − 8.5, − 8.4, and − 8.1 kcal/mol against RdRp (Table 4).

AGP formed four H-bonds with residue Lys621 (2.28 Å), Tyr619 (2.71 Å), Asp760 (2.77 Å), Glu811 (2.95 Å) of RdRp (Fig. 5E, F), while AGP-14/RdRp complex showed five H-bonds with Lys621 (2.28 Å), Ser795 (2.03 Å, 2.41 Å), Lys798 (1.90 Å), and Ser814 (2.28 Å), one hydrophobic, and one C–H bond interactions with residue Pro620 (Fig. 5G, H). Moreover, it also interacted with Ser814 through the C–H bond (Table 5). Two H-bonds with Try617 (2.76 Å) and Asp761 (2.69 Å), two hydrophobic bonds with Lys621 and Arg624, and two electrostatic interactions at Arg553 and Asp623 were noticed for docking of AGP-13 on RdRp. AGP-6 bound at RdRp differently means with different amino acid residue than other AGP derivatives, by three H-bonds Ser795 (2.04 Å), Cys813 (2.84 Å), Cys814 (2.10 Å), and two electrostatic bond interactions (Table 5).

### Binding to NSP15 endoribonuclease

REM and HCQ showed binding energy of − 6.7 and − 5.9 kcal/mol against NSP15. REM formed two H-bonds at Thr341 and Gln245, and six hydrophobic interactions at Trp333 (2 bonds), Val292, Lys345, Leu346, and Tyr343 (Fig. 6A, B). There were three H-bonds at Val276, Lys277, and Asn278, and four hydrophobic interactions at Ile270, Val276, Ile328, and Ile328 were observed for HCQ (Fig. 6C, D). AGP had shown three H-bonds at Gly248 (1.96 Å), Lys290 (2.64 Å), and His235 (2.80 Å), one hydrophobic, and a C–H bond interaction at Tyr343 and Lys345, respectively (Fig. 6E, F). Whereas AGP-15 were showed two H-bonds at Ser294 (2.03 Å, 2.59 Å), three hydrophobic interactions at Lys290, Val315, and Trp333, and a C–H bond interaction at His250 with a binding score of − 8.6 kcal/mol (Fig. 6G, H). However, the binding energy of AGP-10 and three were noticed with − 8.4 and − 8.3 kcal/mol against NSP15.

AGP-3 had shown two H-bonds at Lys290 (2.32 Å, 2.72 Å) and Leu346 (2.81 Å), two hydrophobic interactions at Thr341, His235, and two C–H bonds interaction at Lys345, Glu340 of NSP15 (Table 5). Two H-bonds at Ser294 (1.99 Å, 2.43 Å), two hydrophobic interactions at Trp333, and a C–H bond interaction at His250 were observed for AGP-10 (Table S1).

### Binding to spike glycoprotein

In the current study, REM and HCQ were showed binding energy of − 7.0 and − 5.9 towards S. Seven H-bonds at Gly744, Asn856, Leu977, Arg1000 (2), Phe855, and Asp745, three hydrophobic bond interactions at Val963, Lys964 (2), and three C–H bond interactions at Met740 (C), Val963 (C), and Lys964 (C) were noticed for REM (Fig. 7A, B). Whereas HCQ had two H-bonds at Asp364, Cys336, and one hydrophobic interaction at Trp436, and two C–H bonds interactions at Ala363 (C) and Asp364 (C) (Fig. 7C, D). The co-crystalized compound 2-acetamido-2-deoxy-beta-D-glucopyranose in the crystal structure of S showed interaction with Val-47, Trp436, Asn343, Lys537, Phe133, Glu132, Asn165, Lys129, Cys291, Ser750, Lys-964, Glu988, Arg995, Tyr756, Thr998, and Asn 122.

AGP formed an H-bond with Lys206 (2.06 Å), two hydrophobic interactions with Pro225, Tyr38, and one C–H bond interaction with Phe43 along with binding energy − 7.1 kcal/mol (Fig. 7E, F). With binding energy of − 8.9 kcal/mol, AGP-13 formed two H-bonds with Arg190 (2.43 Å) and His207 (2.51 Å) (Fig. 7G, H), while AGP-15 showed three H-bond formations with Arg190 (2.41 Å, 2.53 Å) and Asn121 (2.45 Å) along with binding energy of − 8.7 kcal/mol. AGP-13 and 15 showed three hydrophobic interactions with Leu226, Val227, and Val126; AGP-15 had hydrophobic interactions with Tyr170 and Phe168. However, AGP-7 has interacted with S through three H-bonds at Arg1000 (1.76 Å, 2.54 Å), Asn856 (2.64 Å), and Leu966 (2.98 Å), and two hydrophobic interactions with Val963 and Leu966 along with binding energy of − 8.4 kcal/mol. Only AGP-7 had shown the interaction with Arg1000, Asn856, and Val963 like REM. AGP-13 and 15 showed interactions with other amino acid residues in the active site of S.

## Discussion

The present study was aimed to identify effective semisynthetic andrographolide derivatives that could interact with various viral proteins and actively reduce or hinder the activity replication of the SARS-CoV-2 virus. Here, we have selected five important target protein of SARS-CoV-2 genome, such as main protease (Mpro, PDB: 6LU7), papain-like protease (PLpro, PDB: 6WUU), spike glycoprotein (S, PDB: 6VXX), NSP15 endoribonuclease (NSP15, PDB: 6VWW), and RNA-dependent RNA polymerase (RdRp, PDB: 6M71). The active site of Mpro contains cysteine and histidine that forms a catalytic dyad, making it an ideal therapeutic target [30]. PLpro is another enzyme responsible for SARS-CoV-2 replication by the action of its catalytic triad (Cys, His, and Asp) and reason for the inflammation of host cells. The viral replication and inflammation of the host cell was reduced by the compounds that bind with catalytic triad [31]. Viruses enter the host cell by binding with host ACE-2 receptor through viral spike protein. The ability of the virus attach to the host cell can be inhibited if spike glycoprotein is inhibited. Endoribonuclease NSP15 is vital for the replication and life cycle of the virus. Hence, we have selected these five enzymes for our current study to explore the interaction efficiency and binding affinity of semisynthetic AGP derivatives on SARS-CoV-2 virus.

The drug-likeness and ADMET properties of 17 AGP derivatives and a two FDA approved drugs (remdesivir and hydroxychloroquine, for comparison purposes only) were predicted using SwissADME and ProTox-II online tools, respectively. Lipinski’s rule of 5 states that a molecule is considered drug-like when it satisfies: molecular weight < 500 Dalton, number of H-bonds donors < 5, number of H-bonds acceptors < 10 and LogP < 5 [32]. Out of 17 AGP derivatives, except compounds 8, 14, 15, and 16, all other derivatives followed Lipinski’s rule of five and were given in Table 1. In that, compounds 8 and 16 has molecular weight > 500, and compounds 14 and 15 has LogP value > 5, but the difference is more negligible. However, the molecular weight of compounds 8 and 16 was marginally lower than REM, and REM also has a high count of hydrogen bond acceptor and rotatable bonds. Hence all the 17 AGP derivatives were considered for docking studies. Only compound 15 showed BBB permeant property like HCQ, and all the AGP derivatives exhibited GI absorption except compounds 6 and 16. Meanwhile, all the 17 AGP derivatives displayed an excellent biological activity score with a value of 0.55, and it is better than the REM value of 0.17 (Table 1). The toxicity prediction showed that all the APG derivatives are free from hepatotoxicity, carcinogenicity, mutagenicity, and cytotoxicity. However, the toxicity prediction results indicated that all the AGP derivatives are immuno-toxic except compound 16. Overall toxicity results indicated that compounds 3, 9, and 17 are safer than other APG derivatives, even though compound 8 showed a better LD_50_ value. The acute oral toxicity for all compounds was estimated as class IV except for two compounds, compound 8 in class VI and 11 in class V. The net outcome of toxicity prediction indicated that all 17 AGP derivatives are safer than REM and HCQ (Table 2).

Lindner et al. (2005) reported that Mpro or NSP5 is a critical enzyme in forming non-structural proteins. It plays a role in the maturation of other nsp and promotes the biosynthesis of the virus. Mpro can be considered a promising target for the treatment of COVID-19 since there is no known Mpro homologous in humans [33]. The co-crystalized compound N3 in the crystal structure of Mpro showed H-bond interaction with His164, Glu166, Gln189, Thr190, Gly143, Phe140, and hydrophobic interaction with His41, Met49, Met165, Leu167, and Pro168. Significant interactions were observed for all the AGP derivatives used in the study than HCQ with catalytic residues and surface accessible residues of Mpro. However, AGP-10 to 17 had shown a better binding score with Mpro than REM. The four amino acid residues involved in the H-bond formation of AGP-11 with Mpro are the same as the residues involved in the H-bond interaction of N3 and REM, which indicates AGP-11 has a greater number of vital H-bond interaction with Mpro than AGP and AGP-16. Both the cyclic hydroxyl groups of AGP were involved in H-bond formation with Mpro; however, both cyclic hydroxyl groups of AGP-16 were not involved. But AGP-16 showed significant hydrophobic interaction with three residues of Mpro as identical as N3 and REM than AGP-11, which had another amino acid in the interaction. Methyl group and the aromatic ring of sulphanilamide substitution of AGP-16 were involved in the hydrophobic interaction. The present study revealed that the most prominent amino acids that formed interactions with the ligand were Thr24, His41, Met49, Gly143, Met165, Glu166, and Gln189.

These AGP derivatives were analyzed based on interactions with vital residues and compared the interactions with reference molecules. AGP-16 and 11 showed more stable H-bonds, hydrophobic, and ionic interactions as compared to reported anti-virals. Our present study results are supported by the study reported by Enmozhi et al. (2020) that the amino acid residues involved in the H-bonds interaction of AGP with Mpro are Gly143, Cys145, and Glu166 [12]. The other studies reported by Kodchakorn et al. (2020) [13] and Lashmi et al. (2020) [14] also supporting our present study result as they had reported that natural AGP analogues interacted with His41, Leu141, Asn142, Gly143, Cys154, Met165, Glu166, Gln189, and Met49.

Once the derivative bound to the active site of Mpro, access to this enzyme, which serves as a critical role in the formation of non-structural proteins and the biosynthesis of the virus, is blocked, which leads to no maturation of other nsp and no biosynthesis of the virus. Stable interaction of all the AGP derivatives with key residues and lowest binding energies indicates the potential for these derivatives to inhibit the activity of Mpro and reduce or prevent viral replication.

PLPro, also known as nsp3, is responsible for viral replication and the host innate immunity [34]. In-depth molecular docking analysis of AGP derivatives with the active sites of PLpro was performed to understand the protein-ligand interactions. The co-crystalized compound peptide inhibitor VIR250 in the crystal structure of PLpro showed H-bond interaction with Gly163, Asp164, Tyr264, Tyr268, and Gly271, and hydrophobic interaction with Ala246, Pro247, Pro248, and Tyr264. Significant interactions were observed for all the AGP derivatives used in the study than HCQ with catalytic residues and surface accessible residues of PLpro. However, AGP, AGP-2, and 14 had shown a better binding score with PLpro than REM. Only two amino acid residues involved in AGP-2 with PLpro are the same as those involved in the interaction of peptide inhibitor VIR250 and REM with PLpro. The carbonyl group of AGP was responsible for the two H-bond interaction with PLpro, although the nitro and carbonyl group of AGP-14 was involved in the H-bond formation with PLpro. Meanwhile, the carbonyl group of AGP-14 was also responsible for the C–H bond interaction with GLU161 residue of PLpro. In comparison, aromatic ring of AGP-14 showed significant hydrophobic interaction with two residues of PLpro as identical as peptide inhibitor VIR250 and REM. The present study revealed that the most prominent amino acids involved in interaction with the ligand were Tyr264, Pro248, Tyr268, and Tyr273. Identified AGP derivatives AGP14 and 2 in the present study showed more stable H-bonds, hydrophobic, and ionic interactions than reported antivirals.

Once the derivative bound to the active site of PLpro, access to this enzyme, which serves as a critical role in replicating the virus, is blocked. The inhibition of PLPro activity by binding to the PLPro catalytic triad can prevent the replication of SARS-CoV-2 and destroy the role of PLPro in the host immune response evasion to reduce the inflammation of host cells [34]. Stable interaction of all the AGP derivatives with key residues and lowest binding energy indicates the potential for these derivatives to inhibit the activity of PLpro and reduce or prevent viral replication. Murugan et al. (2020) reported that the main amino acids involved in the interaction of natural AGP analogues with PLpro areTyr269, Gln270, Gly272, and Tyr274, which support our present study results [16].

SARS-CoV-2 RdRp or non-structural protein 12 (nsp12) is a vital central polymerase involved in RNA replication. It has been studied in various viruses. It is a promising target as studies found that targeting RdRp would not lead to severe toxicity [35]. Elfiky (2020) reported that the active site of RdRp containsTyr618, Cys622, Asn691, Asn695, Met755, Ile756, Leu757, Leu758, Ser759, Asp760, Asp761, Ala762, Val763, Glu811, Phe812, Cys813, and Ser814 residue [5]. The active site key residues are adjacent aspartates, i.e., Asp760 and Asp761 [5, 36]. Other than REM and AGP, AGP-13 and 6 were interacted with the vital residue Asp760 or Asp761, along with the good binding score. But AGP-14 had better binding score than all other AGP derivatives. The cyclic hydroxyl and carbonyl group of AGP was responsible for the H-bond interaction with RdRp; however, the cyclic carbonyl, nitro, and ester groups of AGP-14 were involved in the five H-bond interactions with RdRp. The other common amino acid residues of the active site involved in the AGP interaction with RdRp were Tyr619, Trp617, Lys621, Ser795, Glu811, and Ser814 (Table S1). Identified AGP derivatives AGP-13, 14, and 6 in the present study showed more stable H-bonds, hydrophobic and ionic interactions than reported antivirals.

For a potent RdRp inhibitor, the derivative or compound must interact with key residues in the active site of the RdRp. No new viral genomes are synthesized when the virus’s access to positive sense RNA is blocked by the drug that binds to the RdRP active site. All the identified AGP derivatives, except AGP-3, 4, 5, and 17, have shown stable interaction with key residues, along with the lowest binding energies, which shows the ability of these drugs in inhibition of RdRp, and playing a role in reducing or stopping viral proliferation. Our study results differ from Murugan et al. (2020) as the interaction amino acids with natural AGP analogues reported in their study were Ser644, Arg438, Asp337, Arg509, and Asp508 [16].

Endoribonuclease NSP15 plays a vital role in the replication and life cycle of the virus. The endoribonuclease activity of NSP15 interferes with the innate immune response of the host. All the AGP derivatives and reference compounds have interacted with some of the active site amino acid residues of NSP15: His-235, Asp-240, Ser-242, His-243, Gln-245, Leu-246, Gly-248, His-250, Asn-278, Lys-290, Glu-340, Thr-343, Lys345, and Leu-346 as suggested by Vijayan and Gourinath (2021) [37]. NSP15 is a known anti-viral drug target, and its active site constitutes the catalytic triad His-235, His-250, and Lys-290. All the AGP derivatives except AGP-8, 9, and 13 have interacted with either one or two of the catalytic triad of NSP15. The other essential amino acid residues involved in the active site interaction are Thr-341, Tyr-343, and Ser-294. Identified AGP derivatives AGP-15 and 3 in the present study showed more stable H-bonds, hydrophobic, and ionic interactions than reported anti-viral. Surprisingly the –CH_2_OH group of AGP was involved in three H-bonds formation with NSP15, while the ester group of AGP-15 was responsible for the H-bond interaction with residues of NSP15. However, the methyl substituent at aromatic ring and a cyclic group of AGP-15 were involved in the hydrophobic interaction with NSP15 active site amino acid residues. AGP derivatives binding to the NSP15 active site may potentially block endoribonuclease activity responsible for the protein interference with the innate immune response. These interactions are essential to abolishing viral function and thus reducing the further transmission of the virus. The high binding affinity of AGP derivatives indicates that they can be promising lead compounds as NSP15 inhibitors.

SARS-CoV-2 utilizes the spike protein (S) present on the viral surface to enter the host cells. The protein-protein interaction between the subunits of the spike protein and the ACE-2 receptor active site can be targeted to identify an effective treatment strategy [38]. When spike glycoprotein is inhibited, the ability of the virus to attach to the host cell can be inhibited as well. Identified AGP derivatives AGP-13 and 7 in the present study showed more stable H-bonds and hydrophobic compared to reported antivirals. The ester group of AGP was involved in an H-bond formation and the cyclic ring was responsible for the two hydrophobic interaction with S. However, the aromatic hydroxyl groups of AGP-13 were involved in the two H-bond interactions, and the aromatic ring was responsible for the hydrophobic interaction with S. When AGP derivatives bind to the S active site, spike glycoprotein is inhibited, and the virus's ability to attach to the host cell can also be inhibited. These interactions are essential to abolishing viral function and thus reducing the further transmission of the virus. The high binding affinity of AGP derivatives indicates that they can be promising lead compounds as S inhibitors. Our current study results differ from Lakshmi et al. (2020) [14] and Maurya et al. (2020) [15], since they reported that the natural AGP analogues interacted with Tyr28, Phe59, and Thr761 of spike glycoprotein.

## Conclusion

The absence of a most suitable and active drug for curing COVID-19 has already worsened the pandemic condition. The rapidly changing mechanism and mutation of SARS-CoV-2 made us to think and carry out molecular docking studies of andrographolide derivatives with various essential viral proteins/enzymes to reduce or cure viral infection. Interestingly AGP-12, 14, and 15 showed better binding affinities towards the entire tested enzyme than AGP, REM, and HCQ. AGP derivative 16 had shown − 8.7 kcal/mol binding/docking score for Mpro, AGP-15 showed − 8.6 kcal/mol for NSP15, and AGP-10, 13, and 15 exhibited − 8.7, − 8.9, and − 8.7 kcal/mol, respectively, for S. Overall results indicated that AGP derivatives 14 and 15 could be the best ‘lead’ candidate for the treatment against SARS-CoV-2 infection. However, molecular dynamic studies are essential to confirm the stability of the AGP derivatives 14 and 15 with the SARS-CoV-2 protein complex. The information generated here may provide some insights into exploring potential drugs against SARS-CoV-2 from andrographolide, which can result in the discovery of novel drugs. However, the present study needs further experimental confirmation via in vitro and in vivo studies.

## Supplementary Information

**Additional file 1: Table S1.** Amino acids of protein targets of SARS-CoV-2 involved in interactions with AGP derivatives.

## Data Availability

All the datasets used and/or analyzed during the current study are provided in the article.

## Abbreviations

SARS-CoV-2: Severe acute respiratory syndrome coronavirus-2
COVID-19: Novel coronavirus disease-19
Mpro: Main protease
PLpro: Papain-like protease
S: Spike glycoprotein
NSP15: NSP15 endoribonuclease
RdRp: RNA-dependent RNA polymerase
AGP: Andrographolides
HCQ: Hydroxychloroquine
REM: Remdesivir
