# Supplementary material for: Molecular docking unveils the potential of andrographolide derivatives against COVID-19: an in silico approach
Source: J Genet Eng Biotechnol. 2022 Apr 14;20:58. doi: 10.1186/s43141-022-00339-y (PMC9008396; doi:10.1186/s43141-022-00339-y)
Supplement: Supplementary file 1 — Additional file 1: Table S1. Amino acids of protein targets of SARS-CoV-2 involved in interactions with AGP derivatives. [file 43141_2022_339_MOESM1_ESM.docx]

**Table S1**. Amino acids of protein targets of SARS-CoV-2 involved in interactions with AGP derivatives.

| Bonding interaction | **6LU7** | **6WUU** | **6M71** | **6VWW** | **6VXX** |
| --- | --- | --- | --- | --- | --- |
|  | **Amino acids involved in the interaction** | | | | |
| **HCQ** | | | | | |
| Hydrogen bond | Thr190 | Ala246, Tyr273, Asp302 | Ala840, Phe843, Arg858 | Val276, Lys277, Asn278 | Asp364, Cys336 |
| Hydrophobic | His41, Met49,  Cys145 | Leu162, Tyr264 | Ala547, Ile548, Arg858 | Ile270, Val276, Ile328, Ile328 | Trp436 |
| Others | Glu166 (C) | Asp164 (E), Ser245 (C) | Asp845 (C) |  | Ala363 (C), Asp364 (C) |
| **REM** | | | | | |
| Hydrogen bond | Gly143, Ser144,  Ser144, Cys145,  Glu166, Asn142 | Gly163, Asp164 | Tyr619, Asp760, Trp617 | Thr341, Gln245 | Gly744, Asn856, Leu977, Arg1000, Arg1000, Phe855, Asp745 |
| Hydrophobic | Met165, His41 | Pro248 | Lys798, Lys798 | Trp333, Trp333, Val292, Lys345, Leu346, Tyr343 | Val963, Lys964,  Lys964 |
| Others | Gln189 (C), Glu166 (C) | Asp164 (E), Pro248 (C), Tyr268 (C) | Asp760 (E), Pro620 (C) |  | Met740 (C), Val963 (C), Lys964 (C) |
| **1** | | | | | |
| Hydrogen bond | Gly141, Met165 | Tyr264, Gly271 | Lys621, Tyr619, Asp760, Glu811 | Gly248, Lys290, His235 | Lys206 |
| Hydrophobic |  |  |  | Tyr343 | Pro225, Tyr38 |
| Others |  |  |  | Lys345 (C) | Phe43 (C) |
| **2** | | | | | |
| Hydrogen bond | Thr24, Gly143, His41 | Lys157, Arg166, Arg166, Thr301, Tyr268 | Asn691, Tyr619, Asp760 | Gly248, Lys290 | Asn856, Ser975 |
| Hydrophobic | Met49 |  | Cys622 | Thr341 | Leu966 |
| Others | Thr45 (C) | Ala246 (C), Glu167 (C) | Arg553 (C), Asp623 (C), Asp760 (E) | Lys345 (C), Glu340 (C) | Ser967 (C) |
| **3** | | | | | |
| Hydrogen bond | His41 | Lys157, Arg166, Arg166, Asp164 | Ser795, Glu811 | Lys290, Lys290, Leu346 | Gly339, Cys336 |
| Hydrophobic | Met49, Met49 | Lys157 | Trp800 | Thr341, His235 | Val367 |
| Others | Thr45 (C), Met49 (E), Asp187 (C) | Ala246 (C), Glu167 (C) | Glu811 (E) | Lys345 (C), Glu340 (C) |  |
| **4** | | | | | |
| Hydrogen bond | Thr24, His41 | Arg166, Arg166, Asp164 | Ser795, Glu811 | Lys290 | Arg1000, Arg1000, Phe855 |
| Hydrophobic | Met49 |  | Thr341 |  | Leu966 |
| Others | Thr45 (C) | Glu167 (C) | Glu811 (E) | Lys345 (C) |  |
| **5** | | | | | |
| Hydrogen bond | Gln189 | Asp164 | Ser795, Ser795, Trp800, Lys798 | Gly248, Lys290, Glu340 |  |
| Hydrophobic | His41, Met165 |  |  | Thr341 | Val126 |
| Others | Asn142 (C) | Asn267 (C) | Glu811 (E) | Lys345(C), Lys345 (C) |  |
| **6** | | | | | |
| Hydrogen bond | Gln192, His164 | Arg166, Asp164, Tyr268 | Ser795, Cys813, Ser814 | Gly248, Lys290 | Arg1000 |
| Hydrophobic | Pro168 |  |  | Thr341 | Leu966, Ser967, Leu966 |
| Others | Pro168 (C) | Ala246 (C), Glu167 (C) | Asp761(C), Glu811 (E) | Thr341(C), Lys345(C), Lys345 (C) |  |
| **7** | | | | | |
| Hydrogen bond | Ser46, Cys145 | Arg166, Asp164, Tyr268 | Asp623, Asp760 | Lys290 | Arg1000, Arg1000, Asn856, Leu966 |
| Hydrophobic | Met165, Met49 |  |  | Thr341 | Val963, Leu966 |
| Others | Thr24 (C) | Ala246 (C), Glu167 (C) | Arg553 (C), Asp623 (C), Asp760 (E) | Lys345 (C) |  |
| **8** | | | | | |
| Hydrogen bond | Gly143 | Gly266, Gly163:O | Lys621, Asp761, Asp623 | Thr341, Tyr343, Pro344 | Val42 |
| Hydrophobic | Met165 | Pro247, Pro248 - | Lys621, Arg624,  Tyr455 | Trp333 |  |
| Others |  | Asp164 (C) |  |  |  |
| **9** | | | | | |
| Hydrogen bond | His41 | Asp164, Tyr273 | Arg553, Arg624, Arg624, Asp760, Asp623 | Cys291, Ser294 | Gly744, Leu977, Asn978, Arg1000, Arg1000 |
| Hydrophobic | Met165, His172, Gly143 | Tyr264, Pro247, Pro248 | Arg555 | Tyr343, Lys345,  Leu346 | Val976 |
| Others | Cys145 (S) |  |  |  | Gly744 (C) |
| **10** | | | | | |
| Hydrogen bond | Glu166, Thr190, Gln192 | Arg166 | Ser795, Lys798, Ser814, Asp760 | Ser294, Ser294 | Arg319, Arg319, Gln321 |
| Hydrophobic | Cys145 | Ala246, Pro247, Tyr268, Pro248 | Pro620 | Trp333, Trp333 | Arg319 |
| Others | Pro168 (C), Gln189 (C), Asn142 (C) | Pro248 (C), Asp164 (C), Asp302 (C), Asp164 (C), Met208 (S) | Pro620 (C) | His250 (C) |  |
| **11** | | | | | |
| Hydrogen bond | Gly143, Ser144, Glu166, Gln189 | Arg166, Tyr273, Thr301, Asp302 | Cys813, Ser814 |  | Arg1000 |
| Hydrophobic | Cys145 | Tyr264, Arg166, Met208, Tyr268,  Pro248 | Lys621 | Tyr343, His235,  His243 | Lys964, Leu966,  Val976 |
| Others |  | Pro248 (C),  Ala246 (C), Asp164 (C) | Asp761 (E) | Lys345 (C), Asp240 (C), Gln245 (C) | Phe43 (C), Arg1000 (C) |
| **12** | | | | | |
| Hydrogen bond | Glu166, Thr24 | Tyr268, Tyr273, Asp164 | Cys813, Asp623 | Ser294, Ser294 | Asn282, Leu226 |
| Hydrophobic | Thr25 | Pro247, Pro248 | Pro620 | Trp333, Trp333, Lys290 | Tyr38, Pro225 |
| Others |  |  | Arg553 (C), Lys621 (C), Asp618 (C), Asp623 (C), Asp761 (E), Glu811 (E) | His250 (C) |  |
| **13** | | | | | |
| Hydrogen bond | Thr26, Thr25 | Gly163 | Trp617, Asp761 | Val292 | Arg190, His207 |
| Hydrophobic | Thr25 | Tyr264, Leu162 - | Lys621, Arg624 | Tyr343, Trp333,  Trp333 | Leu226, Val227,  Val126 |
| Others | Met49 (S) |  | Arg553 (E), Asp623 (E) | Asp240 (C) |  |
| **14** | | | | | |
| Hydrogen bond | Thr26 | Leu162, Tyr273 | Lys621, Ser795, Ser795, Lys798, Ser814 |  | Arg190 |
| Hydrophobic |  | Tyr264, Pro248 | Pro620 | Tyr343, His235 | Val126, Leu226,  Val227 |
| Others | Asn142 (C), Thr190 (C), Cys145 (S) | Glu161 (C) | Pro620 (C), Ser814 (C) | Lys345 (C) |  |
| **15** | | | | | |
| Hydrogen bond |  | Thr301 | Lys621, Ser814, Asp761 | Ser294, Ser294 | Arg190, Arg190, Asn121 |
| Hydrophobic | Met49, His41 | Tyr264, Leu162 | Pro620 | Lys290, Val315,  Trp333 | Val126, Leu226,  Val227, Tyr170, Phe168 |
| Others | Gln189 (C) |  | Pro620 (C), Ser814 (C) | His250 (C) |  |
| **16** | | | | | |
| Hydrogen bond | Thr24 | Arg166, Arg166, Asp164 | Cys622 | Ser294, Ser294 |  |
| Hydrophobic | Met49, His41,  Met49, Met165 | Tyr264, Tyr268 | Tyr455, Arg553, Tyr455, Lys621 | Trp333, Trp333,  His235, His235 | Cys291 |
| Others | Met165 (C) | Gln269 (C),  Tyr268 (E), Tyr268 (C) | Arg553 (E), Lys621 (E) | His235 (C), His250 (C) |  |
| **17** | | | | | |
| Hydrogen bond | Gly143 | Tyr273, Asp302 | Arg553, Asp760 | Cys291, Val292, Thr341, His250 | Asp40 |
| Hydrophobic | Pro168 | Tyr264, Pro248,  Arg166 |  | Tyr343, Tyr343,  Lys345, Leu346 |  |
| Others | Met165 (S) | Tyr273 (C), Met208: (S) | Asp623 (E), Cys622 (S) |  |  |

E – Electrostatic, S – pi-sulfur bond, C – carbon-hydrogen bond
